# Comparison of methods for imputing limited-range variables: a simulation study

**DOI:** 10.1186/1471-2288-14-57

**Published:** 2014-04-26

**Authors:** Laura Rodwell, Katherine J Lee, Helena Romaniuk, John B Carlin

**Affiliations:** 1 Clinical Epidemiology and Biostatistics Unit, Murdoch Childrens Research Institute, Flemington Road, Parkville, Melbourne, Victoria 3052, Australia; 2 Department of Paediatrics, Faculty of Medicine, Dentistry and Health Sciences, The University of Melbourne, Melbourne, Australia; 3 Centre for Adolescent Health, Murdoch Childrens Research Institute, Melbourne, Australia

**Keywords:** Multiple imputation, Limited-range, Skewed data, Missing data, Rounding, Truncated regression

## Abstract

**Background:**

Multiple imputation (MI) was developed as a method to enable valid inferences to be obtained in the presence of missing data rather than to re-create the missing values. Within the applied setting, it remains unclear how important it is that imputed values should be plausible for individual observations. One variable type for which MI may lead to implausible values is a limited-range variable, where imputed values may fall outside the observable range. The aim of this work was to compare methods for imputing limited-range variables, with a focus on those that restrict the range of the imputed values.

**Methods:**

Using data from a study of adolescent health, we consider three variables based on responses to the General Health Questionnaire (GHQ), a tool for detecting minor psychiatric illness. These variables, based on different scoring methods for the GHQ, resulted in three continuous distributions with mild, moderate and severe positive skewness. In an otherwise complete dataset, we set 33% of the GHQ observations to missing completely at random or missing at random; repeating this process to create 1000 datasets with incomplete data for each scenario.

For each dataset, we imputed values on the raw scale and following a zero-skewness log transformation using: univariate regression with no rounding; post-imputation rounding; truncated normal regression; and predictive mean matching. We estimated the marginal mean of the GHQ and the association between the GHQ and a fully observed binary outcome, comparing the results with complete data statistics.

**Results:**

Imputation with no rounding performed well when applied to data on the raw scale. Post-imputation rounding and imputation using truncated normal regression produced higher marginal means than the complete data estimate when data had a moderate or severe skew, and this was associated with under-coverage of the complete data estimate. Predictive mean matching also produced under-coverage of the complete data estimate. For the estimate of association, all methods produced similar estimates to the complete data.

**Conclusions:**

For data with a limited range, multiple imputation using techniques that restrict the range of imputed values can result in biased estimates for the marginal mean when data are highly skewed.

## Background

Multiple imputation has become a commonly used and increasingly recommended method for the analysis of incomplete data in observational studies and clinical trials [1,2]. The method involves two stages. First, an imputation procedure is used to predict values for the missing data in multiple copies of the incomplete dataset, thus creating multiple completed datasets. Second, a standard complete-data analysis is conducted on each of these completed datasets, and the results combined to provide overall estimates for the parameters of interest and associated standard errors using Rubin’s rules [3].

The objective when applying the method of multiple imputation to analyse incomplete data is to draw valid inferences while taking account of the missing data [4]. In this study we consider data where the missingness is ignorable. This assumes the data are either missing completely at random (MCAR), that is the missingness does not depend on observed or unobserved data, or are missing at random (MAR), where the probability of a value being missing depends on the observed but not unobserved data. For more information on these definitions and non-ignorable missingness see Schafer and Graham [5]. There are currently two main approaches for imputing data when the missingness mechanism is ignorable. The first of these involves the specification of a multivariate normal (MVN) model for all the variables that are included in the imputation model [6]. The second method is fully conditional specification or multivariate imputation with chained equations (MICE) [7], which requires the specification of a univariate conditional model for each incomplete variable. This latter approach allows for a range of (univariate) imputation models to be specified that closely represent the distributions of the variables with missing data such as linear, logistic, ordinal logistic, poisson, negative binomial and truncated normal regression models.

Even with the more flexible MICE approach, there is a potential mismatch between the assumptions of the imputation model and the distribution of the incomplete variable. This may result in imputed values that are implausible or impossible for the variable. An example where implausible or impossible values can be generated is a limited-range variable.

Limited-range variables are continuous, semi-continuous or ordinal variables that have a restriction to one or both ends of their range. This can either be through the specification of an expected range on a clinical or demographic variable, such as weight or age, where plausible values are determined by the researchers, or it could be a function of the measure itself where there is a restricted range by definition and values outside this range are impossible, such as an ordinal response scale (e.g. a Likert scale), or the sum of a number of items measured on such a scale. While nominal variables, such as race, also can have a defined set of values, there is no meaningful ordering in the potential values. As the methods considered in this study assume an ordered scale we do not include nominal variables in our definition of limited-range variables.

When a limited-range variable is imputed using an MVN or a univariate linear regression model, some of the imputed values may fall outside the range of the variable. While the primary goal of multiple imputation is to obtain valid inferences, and imputed values are not intended to replace the missing data [4], there is some uncertainty as to how to treat imputed values that fall outside the limits of the variable. In the majority of analyses it is possible simply to retain values that fall outside the range, but concern is often expressed that the imputed values themselves should be plausible and that imputation methods should be modified to ensure that imputed values are within the specified range [6,8-10].

There are a number of methods that have been suggested in the literature for handling values that are imputed out of range:

1. **Post-imputation rounding**

One option is to impute the missing values using the standard imputation technique (via MICE or MVN imputation) and then round any values that fall outside the observed or possible range to the limits of the range. This method has been applied in a number of methodological and applied studies (see for example, [10-13]).

The appeal of post-imputation rounding is that, for MVN imputation, this is the only procedure that has been proposed to date that brings the imputed values to within the plausible range. As post-imputation rounding is conducted after the imputed datasets have been created, there are concerns that the rounding of imputed values may cause bias in the resulting parameter estimates, particularly for the marginal mean of the imputed variable, and will inappropriately reduce the variance of the imputed values [14].

2. **Truncated normal regression**

A second option that can be used with univariate imputation or MICE is to impute missing values using truncated normal regression [6]. Under this approach, imputation is carried out using a truncated normal distribution with specified minimum and/or maximum bounds for the incomplete variable in a conditional regression model. The apparent benefit of imputation using truncated normal regression is that the restriction of the range is specified within the imputation procedure, so there are no post-imputation adjustments made to the imputed data values, unlike the post-imputation rounding procedure. A possible issue associated with this method is that it assumes an underlying normal distribution and may be sensitive to skewness in the data.

3. **Predictive mean matching**

Available when performing univariate regression imputation or MICE for a continuous variable, the method of predictive mean matching is a partially parametric approach that first predicts the values for the missing data using a linear prediction model. For each missing value, the observed value, or *k* observed values that are closest to the predicted mean of the missing value are selected. If *k =* 1, this observed value is used to replace the missing observation; if *k >* 1 then an observed value is randomly selected from the *k* nearest candidates [15]. The main attraction of this method is that, as only observed values are used, the distribution and range of the data are preserved and plausible imputed values are guaranteed.

Another issue that arises when imputing missing values is how to impute variables that are non-normally distributed, since both MICE and MVN imputation assume conditional normality for imputing continuous variables. One option that is commonly used in practice to handle such variables to make the normality assumption more plausible is to apply a de-skewing transformation, such as the log or zero-skewness log transformation, prior to imputation [13]. The process of transforming a variable prior to imputation has been applied in conjunction with a number of the methods above, such as post-imputation rounding [13]. One issue that arises with using a de-skewing transformation such as the log transformation for positively skewed data is that when the imputed values are transformed back to the original scale, the imputed values can have very large outlying values [14].

A recent paper by von Hippel [14] focused on the imputation of non-normal data and compared the methods of rounding, truncating and transformation when imputing skewed variables with a lower bound. Von Hippel recommended that imputation be carried out on the raw scale with no transformation or post-imputation rounding, regardless of whether the data are normally distributed or not. His focus however, was fairly restrictive as he only considered data from an exponential distribution with the lower range restricted.

Given the limited comparison of methods for handling limited-range variables to date, we undertook a systematic study into the imputation of such variables. We focus on scenarios where both upper and lower limits are restricted, as well as examining data from a range of skewed distributions. We consider imputation using univariate linear regression and allowing imputed values to fall outside the range or rounding the values after imputation (post-imputation rounding); imputing with a truncated normal regression model; and imputation via predictive mean matching. We apply each of these methods on the raw and transformed scales of an incomplete variable with varying amounts of skewness using a simulation study in which we redraw missingness from a real data example with complete data.

## Methods

### Sample

The Victorian Adolescent Health Cohort Study (VAHCS) is a repeated measures cohort study of health in adolescents (waves 1 to 6) and young adults (waves 7 to 9), which was conducted between 1992 and 2008. The original sample of 1943 participants was randomly sampled from schools in Victoria, Australia, when they were aged 14 – 15 years. Data collection protocols were approved by The Royal Children’s Hospital’s Ethics in Human Research Committee. For further details on the cohort, see Reference [16].

### Target analysis

The target analysis in the current study was a summary of minor psychiatric illness, measured by the General Health Questionnaire (GHQ) [17] at wave 8 (age approximately 24 years), and the association between GHQ at wave 8 and the likelihood of a person continuing to live in the family home at wave 9 (at approximately 29 years).

The exposure of interest, the GHQ, is a 12-item questionnaire that was developed to measure minor psychiatric illness in the community [17]. Each of the 12 items in the GHQ screens for a symptom that is indicative of psychological distress and has four response options that reflect the increasing degree to which the participant has experienced the symptom. An example of a question in this scale is: “Have you lost much sleep over worry?” with the possible responses being: “not at all/no more than usual/rather more than usual/much more than usual”.

The GHQ can be scored using three different methods, as described by Donath [18]: the Likert, standard and C-GHQ scores. The Likert scoring method (possible range 0 – 36) has a scoring pattern of 0-1-2-3 for each of the items, with 3 representing the most extreme presence of the symptom. The total of the Likert scores provides a measure of the severity of psychological distress. The standard scoring method (possible range 0 – 12) has a scoring pattern of 0-0-1-1 for each item, with the last two responses indicating presence of the symptom and the total measures psychological distress using a count of the number of items that have a positive response. The C-GHQ scoring (possible range 0 – 12) is an adaptation of the standard scoring method, with the positively worded items scored 0-0-1-1 as in the standard scoring method, and the negatively worded items, such as the example above, allocated a scoring pattern of 0-1-1-1. This latter approach was developed to capture the possible presence of symptoms associated with the response “no more than usual” [19].

The outcome of interest in the target analysis was a binary indicator of whether the participants lived at their parent(s)’ home at wave 9, as determined from a direct question in the questionnaire administered at this wave.

A third variable used in this simulation study was GHQ measured at wave 9; this was a four-level categorical variable derived from the Likert scoring method, with categories of 0–5 (low), 6–8 (moderate), 9–11 (high) and 12–36 (very high). This variable was included as a complete auxiliary variable in the imputation model, as it is correlated with GHQ score at wave 8. To ensure that variation in the scaling of this auxiliary variable did not confound the results, the same categorical variable for GHQ at wave 9 was used in all imputation models, regardless of the scoring method of GHQ at wave 8.

For the sake of this paper we restricted our analysis to females with complete data on the exposure, outcome and auxiliary variable, resulting in a sample size of 714 participants.

Due to the steps involved in the simulation study (described below), particularly the reduction of the dataset to complete data and the omission of key confounders from the analysis, reported results are not intended to realistically address the substantive question about association between mental health and living at home in young adulthood.

### Simulation method

The method for this simulation study is based on that used by Brand et al. [20] and described further by van Buuren [21]. We start with a sample that have complete data and simulate the missing data process by repeatedly setting a proportion of the data to missing.

We examined the imputation methods under both MCAR and MAR missingness conditions. For MCAR, missing values were randomly imposed for approximately 33% of values in the GHQ at wave 8. For the MAR condition, values were set to missing depending on the binary outcome (living at home at wave 9) and 4-level ordinal auxiliary variable (GHQ at wave 9), with a probability determined by the logistic regression model:

(1) $$\begin{array}{ll} \text{logit}\mathrm{Pr}\left(\text{missing}\right)= & \alpha +\beta_{1}\text{Living}+\beta_{2}\text{GHQ}9_{\text{moderate}}\\ & +\beta_{3}\text{GHQ}9_{\text{high}}+\beta_{4}\text{GHQ}9_{\text{very}\;\text{high}} \end{array}$$

where Living is an indicator of living at home at wave 9 and GHQ9_*moderate*_, GHQ9_*high*_ and GHQ9_*very high*_ represent indicators for moderate, high and very high GHQ at wave 9. We fixed the coefficients of this logistic regression to be *β*_1_ = 1.25 (corresponding to an odds ratio [OR] of 3.5),  *β*_2_ = 0.2 (OR = 1.22), *β*_3_ = 0.3 (OR = 1.35) and *β*_4_ = 0.4 (OR = 1.5), which represent modest but potentially realistic relationships between these variables and missingness. The value of *α*  was chosen empirically in order to produce missing values in approximately 33% of cases.

For each scoring method of the GHQ at wave 8 and both missingness scenarios, we conducted the following steps *N* = 1000 times:

• Missingness was generated in the complete dataset as described above.

• The following imputation methods were used with *m* = 20 imputations performed for each procedure:

– Linear regression imputation (applied using the Stata command: mi impute regress) with no post-imputation rounding.

– Linear regression imputation with post-imputation rounding, with the limits specified as 0 (min) and 12 (max) for the C-GHQ and standard scoring and 0 (min) and 36 (max) for the Likert scoring.

– Truncated normal regression (carried out using mi impute truncreg), with the lower and upper limits specified as the same limits used for the post-imputation rounding method.

– Predictive mean matching (carried out using mi impute pmm), with the number of nearest neighbour candidates specified as *k* =5 [22].

• For all imputation analyses, imputation models included the complete outcome variable (living at home at wave 9) and the complete auxiliary variable (GHQ at wave 9, included as a 4-level ordinal variable).

• Each of the above methods was also applied to the incomplete GHQ variable transformed using a shifted log transformation (using the lnskew0 in Stata version 13 [23]). Where relevant, the minimum and maximum limits were specified on the shifted log scale to be equivalent to those on the raw scale.

• Target parameters of interest for evaluation of the imputation approaches were the marginal mean of the GHQ at wave 8 and the log odds of living at home at wave 9 given GHQ score at wave 8.

### Performance measures for evaluating different methods

In order to evaluate these various imputation approaches, we compared our estimated statistics from the simulations to the complete data statistics.

Using the notation of Brand et al. [20] and van Buuren [21], we define *Q* to be the unknown population parameter of interest, for which we have a complete data point estimate, denoted Q^. For each imputation method within one simulated dataset, we obtain an average point estimate across the *m* imputed datasets, which we denote ${\overset{¯}{Q}}_{m}$. In this simulation design, we consider $\hat{Q}$ to be both an estimate of *Q* and an estimand for ${\overset{¯}{Q}}_{m}$. Since we are fixing Q^ the performance measures we consider relate to the properties of ${\overset{¯}{Q}}_{m}$ under repeated sampling of the missingness (assuming that Q^ is a valid estimate of  *Q* under repeated sampling of the complete data). We calculated bias (in the restricted sense described) by comparing the average of ${\overset{¯}{Q}}_{m}$ over our 1000 simulated datasets ($E\left[{\overset{¯}{Q}}_{m}\right]$) with the complete data estimate (Q^):

(2) Bias=EQ¯m-Q^

To assess the variance estimates from the various imputation approaches under this simulation design, Brand et al. [20] distinguished between the two components of variance, within-imputation and between-imputation, that are estimated and pooled using Rubin’s rules [3] to estimate the total variance of ${\overset{¯}{Q}}_{m}$ as an estimate of *Q*. The *within-imputation* variance (${\overset{¯}{U}}_{m}$), which is the average of the square of the standard errors of the point estimates derived from each of the *m* imputed datasets, should produce an unbiased estimate (over repeated sampling of the missingness) of the complete-data variance estimate, denoted *U*:

(3) $$E\left[{\overset{¯}{U}}_{m}\right]=U$$

As with the bias measure, we assess the performance of the within-imputation variance estimates by averaging ${\overset{¯}{U}}_{m}$ across the 1000 simulations and comparing the result with *U*.

The second component of variance is the *between-imputation* variance, which represents the variability due to missing data [21], and is estimated by *B*_*m*_, the empirical variance of the *m* estimates of *Q*  obtained across the imputed datasets. On average, this quantity should estimate the actual variability observed in the MI point estimates across the repeated draws of the missingness ($i.e.\text{Var}\left({\overset{¯}{Q}}_{m}\right)$) so that the following condition should hold:

(4) $$\text{Var}\left({\overset{¯}{Q}}_{m}\right)=\left(1+m^{-1}\right)E\left[B_{m}\right]$$

We therefore assess the performance of the between-imputation variance *B*_*m*_ in estimating the actual variability of the estimates ${\overset{¯}{Q}}_{m}$.

The final measure of performance we used is a coverage property based on the proportion of (nominal) 95% confidence intervals that contain Q^, the point estimate from the complete data, over repeated draws of the missingness, which we estimate as:

(5) P[Q¯m-1+m-1Bmtm-1;0.975)≤Q^≤(Q¯m+1+m-1Bmtm-1;0.975]

This coverage proportion should equal 0.95, with both under and over coverage indicating a problem.

We considered each of the above evaluations of performance for the estimates of the marginal mean of the GHQ measure at wave 8 and the log odds ratio for the association between living at home at wave 9 and GHQ score at wave 8.

## Results

**Figure 1:**
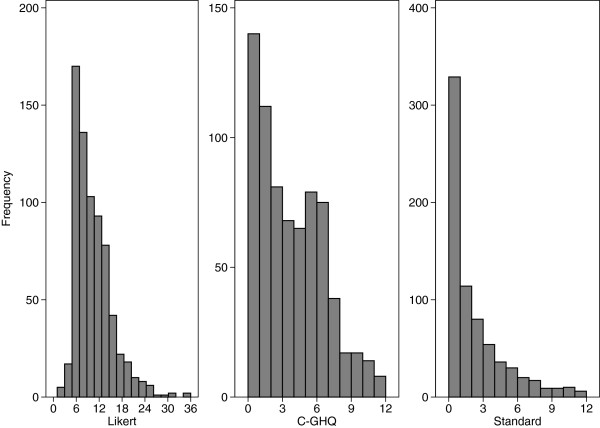
Distribution of complete data for three scoring methods of GHQ at wave 8 (714 females).

Figure 1 shows the distributions of the three GHQ scoring methods. The Likert score has a weak positive skew; the C-GHQ scores has a moderate positive skew, and the standard scores method data has a point mass at zero and a severe positive skew.

Table 1 presents the average percentage of values imputed outside the range of the GHQ under the 3 different scoring methods, and on the raw and transformed scales using univariate linear regression with no rounding, across the 1000 simulated datasets. We observe that on the raw scale, for both MCAR and MAR, as the variable became more positively skewed, a greater percentage of values were imputed below the lower limit of the specified range. This pattern was similar for the transformed scales but there was a higher percentage of values imputed above the upper bound.

**Table 1:** Average percentage of missing values imputed outside the specified range using linear regression imputation, by scoring method

	**GHQ raw scale**				**GHQ transformed**			
	**% imputed below lower bound**		**% imputed above upper bound**		**% imputed below lower bound**		**% imputed above upper bound**	
**Scoring method**	**MCAR**	**MAR**	**MCAR**	**MAR**	**MCAR**	**MAR**	**MCAR**	**MAR**
Likert	2.0	2.0	0.0	0.0	0.0	0.0	0.2	0.2
C-GHQ	12.2	11.6	0.1	0.1	7.9	7.4	1.8	0.2
Standard	23.0	24.0	0.0	0.0	14.3	14.9	6.5	7.5

Note: Mean number of missing values per dataset is 238.

### Marginal mean

Table 2 presents the results for estimation of the marginal mean when the imputation methods were applied to the GHQ data without transformation.

**Table 2:** Performance measures for the estimation of the marginal mean, GHQ scores on raw scale

**Scenario**	**Validation statistics**					
**Likert**	Q^=10.2311		***U*** = 0.1855			
**MCAR**	$E\left[{\overset{¯}{Q}}_{m}\right]$	**bias**	$E\left[{\overset{¯}{U}}_{m}\right]$	$\text{Var}\left({\overset{¯}{Q}}_{m}\right)$	(1 + ***m***^- 1^)***E***[***B***_***m***_]	**coverage for** Q^
Regression, non-rounded	10.2319	0.0008	0.1862	0.0179	0.0174	0.943
Post-imputation rounding	10.2445	0.0134	0.1851	0.0180	0.0167	0.941
Truncated regression	10.2206	-0.0105	0.1846	0.0172	0.0172	0.959
Predictive mean matching	10.1805	-0.0506	0.1832	0.0176	0.0138	0.894
**MAR**						
Regression, non-rounded	10.2243	-0.0068	0.1838	0.0220	0.0191	0.939
Post-imputation rounding	10.2353	0.0042	0.1828	0.0221	0.0185	0.928
Truncated regression	10.2183	-0.0128	0.1825	0.0219	0.0191	0.936
Predictive mean matching	10.1378	-0.0933	0.1818	0.0213	0.0158	0.852
**C-GHQ**	Q^=3.3179		***U*** = 0.1055			
**MCAR**	$E\left[{\overset{¯}{Q}}_{m}\right]$	**bias**	$E\left[{\overset{¯}{U}}_{m}\right]$	$\text{Var}\left({\overset{¯}{Q}}_{m}\right)$	(1 + ***m***^- 1^)***E***[***B***_***m***_]	**coverage for** Q^
Regression, non-rounded	3.3178	-0.0002	0.1058	0.0055	0.0054	0.956
Post-imputation rounding	3.3741	0.0561	0.1023	0.0053	0.0044	0.846
Truncated regression	3.5582	0.2402	0.1025	0.0054	0.0077	0.233
Predictive mean matching	3.2931	-0.0248	0.1048	0.0058	0.0050	0.925
**MAR**						
Regression, non-rounded	3.3150	-0.0029	0.1055	0.0065	0.0061	0.946
Post-imputation rounding	3.3687	0.0508	0.1021	0.0061	0.0050	0.880
Truncated regression	3.5505	0.2326	0.1024	0.0060	0.0086	0.318
Predictive mean matching	3.2664	-0.0515	0.1045	0.0067	0.0059	0.880
**Standard**	Q^=1.8081		***U*** = 0.094			
**MCAR**	$E\left[{\overset{¯}{Q}}_{m}\right]$	**bias**	$E\left[{\overset{¯}{U}}_{m}\right]$	$\text{Var}\left({\overset{¯}{Q}}_{m}\right)$	(1 + ***m***^- 1^)***E***[***B***_***m***_]	**coverage for** Q^
Regression, non-rounded	1.8085	0.0004	0.0951	0.0045	0.0045	0.950
Post-imputation rounding	1.9276	0.1195	0.0894	0.0046	0.0029	0.436
Truncated regression	2.2988	0.4907	0.0982	0.0078	0.0434	0.293
Predictive mean matching	1.7791	-0.0290	0.0934	0.0044	0.0035	0.894
**MAR**						
Regression, non-rounded	1.8057	-0.0024	0.0941	0.0056	0.0051	0.933
Post-imputation rounding	1.9198	0.1117	0.0887	0.0054	0.0033	0.552
Truncated regression	2.3043	0.4962	0.0984	0.0082	0.0445	0.291
Predictive mean matching	1.7624	-0.0457	0.0930	0.0053	0.0040	0.848

*Key*: Q^ = complete data estimate; *U* = estimated variance of Q^ from complete data; $E\left[{\overset{¯}{Q}}_{m}\right]$ = average of MI-based point estimates across 1000 simulated datasets; bias = difference between $E\left[{\overset{¯}{Q}}_{m}\right]$ and Q^; $E\left[{\overset{¯}{U}}_{m}\right]$ = average of estimated within-imputation variance across simulated datasets; $\text{Var}\left({\overset{¯}{Q}}_{m}\right)$ = variance of the MI point estimates across simulated datasets; (1 + m^- 1^)E[B_m_] = average of estimated between-imputation variance (with adjustment for number of imputations) across simulated datasets; coverage = proportion of (nominally) 95% confidence intervals that contain the complete data estimate.

For the scoring of the GHQ based on the Likert scale, which was only mildly skewed, all of the MI-based point estimates $\left(E\left[{\overset{¯}{Q}}_{m}\right]\right)$ were on average close to the complete data estimate Q^*,* reflecting minimal bias in the estimates. The average within-imputation variance estimates $\left(E\left[{\overset{¯}{U}}_{m}\right]\right)$ were also close to the complete-data variance *U* for all approaches. Considering the between-imputation variance ((**1** + **1**/***m***)***E***[***B***_***m***_])*,* although the non-rounded regression and truncated regression methods produced estimates close to the variance of ${\overset{¯}{Q}}_{m}$ across the 1000 replications, post-imputation rounding and predictive mean matching both produced under-estimates of the actual between-imputation variance. For the MCAR scenario for the GHQ Likert scoring, with the exception of predictive mean matching, the methods had a coverage proportion close to 0.95. Predictive mean matching had coverage of 0.894, reflecting a small bias in the estimate and under-estimation of the between-imputation variance. For the MAR scenario, all methods had a slight under-coverage of the complete data estimate, with predictive mean matching again having the lowest coverage.

For the moderately skewed measure, the C-GHQ, imputation based on linear regression with no rounding had the least biased point estimate under both the MCAR and MAR scenarios. Combined with the estimated between-imputation variance closely reflecting the variance of ${\overset{¯}{Q}}_{m}$*,* this resulted in coverage proportions close to 0.95 for the unrounded approach in both scenarios. Imputation based on truncated normal regression, and to a lesser extent post-imputation rounding, produced point estimates that were on average higher than the complete data estimate. The coverage proportions for these two methods were low, in particular for truncated regression. Predictive mean matching also produced a slight under-coverage of the complete data estimate.

The most severely skewed standard scoring method displayed a very similar pattern of results to those of the C-GHQ. The non-rounded regression method resulted in estimates with low bias and the within- and between-imputation variance estimates performed well. This resulted in coverage close to 0.95 for the MCAR condition and only slight under-coverage under the MAR condition. Both the post-imputation rounding and truncated regression imputation methods produced biased estimates and poor coverage. Predictive mean matching produced an average point estimate close to that of the complete data, but, consistent with the previous observations for both the Likert and C-GHQ, the estimated between-imputation variance was low, resulting in under-coverage.

Table 3 presents results for the marginal mean with the imputation procedures applied following transformation to the shifted log scale.

**Table 3:** Performance measures for the estimation of the marginal mean with transformed GHQ scores

**Scenario**	**Validation statistics**					
**Likert**	Q^=10.2311		***U*** = 0.1855			
**MCAR**	$E\left[{\overset{¯}{Q}}_{m}\right]$	**bias**	$E\left[{\overset{¯}{U}}_{m}\right]$	$\text{Var}\left({\overset{¯}{Q}}_{m}\right)$	(1 + ***m***^- 1^)***E***[***B***_***m***_]	**coverage for** Q^
Regression, non-rounded	10.2366	0.0055	0.1861	0.0181	0.0174	0.947
Post-imputation rounding	10.1446	-0.0865	0.1857	0.0416	0.0170	0.820
Truncated regression	10.2227	-0.0084	0.1840	0.0181	0.0163	0.935
Predictive mean matching	10.1926	-0.0385	0.1837	0.0174	0.0148	0.916
**MAR**						
Regression, non-rounded	10.2119	-0.0192	0.1846	0.0223	0.0197	0.928
Post-imputation rounding	10.0985	-0.1326	0.1842	0.0628	0.0193	0.758
Truncated regression	10.2010	-0.0301	0.1825	0.0217	0.0180	0.915
Predictive mean matching	10.1401	-0.0910	0.1819	0.0216	0.0164	0.858
**C-GHQ**	Q^=3.3179		***U*** = 0.1055			
**MCAR**	$E\left[{\overset{¯}{Q}}_{m}\right]$	**bias**	$E\left[{\overset{¯}{U}}_{m}\right]$	$\text{Var}\left({\overset{¯}{Q}}_{m}\right)$	(1 + ***m***^- 1^)***E***[***B***_***m***_]	**coverage for** Q^
Regression, non-rounded	3.3268	0.0088	0.1087	0.0057	0.0063	0.960
Post-imputation rounding	3.3231	0.0051	0.1053	0.0058	0.0052	0.931
Truncated regression	3.5563	0.2384	0.1028	0.0053	0.0048	0.096
Predictive mean matching	3.3009	-0.0170	0.1049	0.0057	0.0053	0.941
**MAR**						
Regression, non-rounded	3.3265	0.0086	0.1094	0.0065	0.0077	0.969
Post-imputation rounding	3.3185	0.0005	0.1055	0.0065	0.0063	0.954
Truncated regression	3.5452	0.2273	0.1027	0.0060	0.0055	0.160
Predictive mean matching	3.2722	-0.0457	0.1045	0.0069	0.0059	0.886
**Standard**	Q^=1.8081		***U*** = 0.0947			
**MCAR**	$E\left[{\overset{¯}{Q}}_{m}\right]$	**bias**	$E\left[{\overset{¯}{U}}_{m}\right]$	$\text{Var}\left({\overset{¯}{Q}}_{m}\right)$	(1 + ***m***^- 1^)***E***[***B***_***m***_]	**coverage for** Q^
Regression, non-rounded	263.63	261.82	30917	53900000	998000000	0.992
Post-imputation rounding	1.7996	-0.0086	0.1055	0.0037	0.0074	0.992
Truncated regression	2.2661	0.4580	0.0953	0.0056	0.0047	0.000
Predictive mean matching	1.8052	-0.0029	0.0943	0.0043	0.0041	0.940
**MAR**						
Regression, non-rounded^Ɨ^						
Post-imputation rounding	1.8035	-0.0046	0.1074	0.0043	0.0095	0.989
Truncated regression	2.2603	0.4522	0.0951	0.0061	0.0054	0.000
Predictive mean matching	1.7829	-0.0252	0.0937	0.0053	0.0045	0.909

^Ɨ^Values larger than those obtained under the MCAR condition *Key*: Q^ = complete data estimate; *U =* estimated variance of Q^ from complete data; $E\left[{\overset{¯}{Q}}_{m}\right]$ = average of MI-based point estimates across 1000 simulated datasets; bias = difference between $E\left[{\overset{¯}{Q}}_{m}\right]$ and Q^; $E\left[{\overset{¯}{U}}_{m}\right]$ = average of estimated within-imputation variance across simulated datasets; $\text{Var}\left({\overset{¯}{Q}}_{m}\right)$ = variance of the MI point estimates across simulated datasets; (1 + m^- 1^)E[B_m_] = average of estimated between-imputation variance (with adjustment for number of imputations) across simulated datasets; coverage = proportion of (nominally) 95% confidence intervals that contain the complete data estimate.

For the Likert scoring, there was low bias in the point estimate across the imputation methods, except for post-imputation rounding under MAR. Imputation using regression with no rounding produced the best coverage for both the MCAR and MAR conditions.

Imputation using regression with no rounding applied to the transformed C-GHQ outcome produced low bias in the point estimate but slightly overestimated within- and between-imputation variances, resulting in slight over-coverage. The high estimated variance appears to have been the result of a small number of high values imputed on the log–scale. Post-imputation rounding performed well in this scenario, in terms of both bias and coverage. Predictive mean matching resulted in low bias for both MCAR and MAR, but for the MAR condition produced a slight under-coverage of the complete data estimate. Truncated regression produced biased point estimates in both MCAR and MAR scenarios and the estimated within- and between- imputation variances were low, resulting in drastic under-coverage.

Performance of the imputation methods was particularly erratic when applied to the transformed version of the standard scale, which had an extreme skew on the raw scale, particularly for the non-rounded imputation method. For this method, there was very high bias in the estimates, due to some very high imputed values. When these values were rounded using post-imputation rounding, the point estimates were less biased, but the variance was high, resulting in over-coverage. Consistent with the results for the C-GHQ, truncated regression was biased and underestimated the variance resulting in none of the estimated confidence intervals covering the point estimate from the complete data in either the MCAR or MAR scenario. Particularly for MCAR, predictive mean matching performed well for this variable, compared with the other imputation methods.

### Regression coefficient

Given the generally poor results for estimation of the marginal mean using the transformed GHQ scores we only consider results for the regression coefficient using the GHQ scores without transformation. Results for the transformed GHQ scores are given in the Additional file 1.

Results for estimation of the regression coefficient for the association between GHQ at wave 8 and living at home at wave 9 (Table 4) were similar for all imputation approaches across the scoring methods (Likert, C-GHQ and standard) and both missingness conditions (MCAR and MAR); we observed low bias across all of the average point estimates, but there was a slight over-estimation of the within-imputation variance and an under-estimation of the between-imputation variance. Using regression with no post-imputation rounding produced the coverage closest to 0.95 in all scenarios, apparently due to better estimation of the between-imputation variance compared with the other approaches.

**Table 4:** Performance measures for the estimation of the regression coefficient with GHQ scores on raw scale

**Scenario**	**Validation statistics**					
**Likert**	Q^=0.03227		***U*** = 0.02143			
**MCAR**	$E\left[{\overset{¯}{Q}}_{m}\right]$	**bias**	$E\left[{\overset{¯}{U}}_{m}\right]$	$\text{Var}\left({\overset{¯}{Q}}_{m}\right)$	(1 + ***m***^- 1^)***E***[***B***_***m***_]	**coverage of** Q^
Regression, non-rounded	0.03181	-0.00046	0.02211	0.00027	0.00022	0.922
Post-imputation rounding	0.03192	-0.00035	0.02219	0.00027	0.00022	0.920
Truncated regression	0.03247	0.00020	0.02217	0.00028	0.00022	0.914
Predictive mean matching	0.02551	-0.00676	0.02223	0.00019	0.00016	0.918
**MAR**						
Regression, non-rounded	0.02888	-0.00340	0.02270	0.00080	0.00067	0.927
Post-imputation rounding	0.02926	-0.00301	0.02279	0.00079	0.00066	0.924
Truncated regression	0.03010	-0.00217	0.02278	0.00084	0.00066	0.926
Predictive mean matching	0.01727	-0.01500	0.02298	0.00036	0.00036	0.911
**C-GHQ**	Q^=0.04794		***U*** = 0.03967			
**MCAR**	$E\left[{\overset{¯}{Q}}_{m}\right]$	**bias**	$E\left[{\overset{¯}{U}}_{m}\right]$	$\text{Var}\left({\overset{¯}{Q}}_{m}\right)$	(1 + ***m***^- 1^)***E***[***B***_***m***_]	**coverage of** Q^
Regression, non-rounded	0.04694	-0.00100	0.04014	0.00077	0.00076	0.946
Post-imputation rounding	0.04805	0.00012	0.04123	0.00080	0.00069	0.932
Truncated regression	0.04360	-0.00433	0.04130	0.00086	0.00073	0.925
Predictive mean matching	0.03738	-0.01055	0.04033	0.00053	0.00056	0.939
**MAR**						
Regression, non-rounded	0.04283	-0.00511	0.04056	0.00232	0.00219	0.939
Post-imputation rounding	0.04932	0.00138	0.04158	0.00224	0.00195	0.928
Truncated regression	0.06470	0.01676	0.04133	0.00243	0.00210	0.906
Predictive mean matching	0.02442	-0.02352	0.04087	0.00109	0.00121	0.929
**Standard**	Q^=0.05236		***U*** = 0.04202			
**MCAR**	$E\left[{\overset{¯}{Q}}_{m}\right]$	**bias**	$E\left[{\overset{¯}{U}}_{m}\right]$	$\text{Var}\left({\overset{¯}{Q}}_{m}\right)$	(1 + ***m***^- 1^)***E***[***B***_***m***_]	**coverage of** Q^
Regression, non-rounded	0.05066	-0.00170	0.04336	0.00101	0.00085	0.938
Post-imputation rounding	0.05252	0.00016	0.04550	0.00106	0.00067	0.889
Truncated regression	0.04613	-0.00623	0.04218	0.00101	0.00096	0.930
Predictive mean matching	0.04069	-0.01167	0.04368	0.00065	0.00061	0.929
**MAR**						
Regression, non-rounded	0.04557	-0.00679	0.04441	0.00278	0.00261	0.942
Post-imputation rounding	0.06226	0.00990	0.04590	0.00238	0.00187	0.911
Truncated regression	0.09485	0.04249	0.04092	0.00233	0.00259	0.857
Predictive mean matching	0.02669	-0.02567	0.04517	0.00122	0.00139	0.939

*Key*: Q^ = complete data estimate; *U* = estimated variance of Q^ from complete data; $E\left[{\overset{¯}{Q}}_{m}\right]$ = average of MI-based point estimates across 1000 simulated datasets; bias = difference between $E\left[{\overset{¯}{Q}}_{m}\right]$ and Q^; $E\left[{\overset{¯}{U}}_{m}\right]$ = average of estimated within-imputation variance across simulated datasets; $\text{Var}\left({\overset{¯}{Q}}_{m}\right)$ = variance of the MI point estimates across simulated datasets; [(1 + m^- 1^)E[B_m_] = average of estimated between-imputation variance (with adjustment for number of imputations) across simulated datasets; coverage = proportion of (nominally) 95% confidence intervals that contain the complete data estimate.

## Discussion

In this study we compared a range of methods for imputing limited-range variables with varying amounts of skewness, with and without applying a de-skewing transformation prior to imputation. We found the performance of the methods differed depending on the degree of skewness and the target estimate of interest. While we saw evidence of some bias and under-coverage of the complete data estimate when estimating the marginal mean from some of the imputation approaches, estimating an association was more robust to the imputation approach used, particularly when data were imputed on the raw scale.

The best performance for estimation of the marginal mean was obtained using linear regression imputation on the raw scale with no rounding, for all degrees of skewness. Although some values were imputed outside the range of possible values, this method had a low bias and estimated the within- and between- imputation variance adequately, resulting in generally good coverage across repeated sampling of the missingness.

Using post-imputation rounding introduced some bias in the estimate of the marginal mean and this increased as the number of values imputed below the minimum value increased. This bias, coupled with a general under-estimation of the between-imputation variance from the post-imputation rounding method, resulted in under-coverage, particularly for the standard GHQ with the severe skew. This finding is consistent with the results reported by von Hippel [14]. Although examining a rather different scenario, Horton [24] also presented evidence that rounding imputed values of binary variables to ensure plausible values following normal imputation may introduce bias in the parameter estimate of interest, which in Horton’s example was a Bernoulli probability of success.

In the current paper, imputation with the truncated normal regression model was also found to induce bias when estimating the marginal mean for the scenario of moderately and extremely skewed data. Similarly, von Hippel [14] found truncated normal regression resulted in biased inference. For data with a weak skew, the truncated regression method performed well, particularly in the MCAR scenario.

For the estimate of the marginal mean, predictive mean matching produced coverage proportions that were lower than those produced by the method of imputation with no rounding. This was due to a combination of a slight bias in the estimates as well as low estimated between-imputation variance. The observed under-coverage may be partly due to the matching algorithm used by -mi impute pmm- [22].

The results for estimation of the regression coefficient were less sensitive to the choice of imputation method when imputation was carried out on the raw scale, with a low bias observed across all methods. Across all conditions, the coverage was closest to 0.95 when linear regression with no post-imputation rounding was used.

Although transforming the variables prior to imputation resulted in fewer values imputed outside the range than imputing on the raw scale, this did not provide any additional benefit for the Likert and C-GHQ scoring conditions compared with carrying out imputation on the raw scale. It did, however, result in some very large outliers among the imputed values when applied to the standard scoring method, the method with the largest skew, therefore limiting the appeal of transforming data prior to imputation.

One method of imputation that we did not consider was ordinal logistic regression [25]. While this method would preserve the range of the variable, it was not considered here due to the large number of categories for the GHQ variable being imputed; for example, in the case of the Likert scoring of the GHQ with a range of 0 – 36, there are 37 ordinal categories. It is possible that imputation methods based on ordinal logistic regression, or similar methods for imputing ordinal data would perform well for ordinal variables with a small number of categories.

Our evaluation of alternative approaches to imputation for limited-range variables was conducted within a simulation framework in which we fixed a particular complete dataset of interest and repeatedly set data to missing, comparing the results of MI-based inferences for two target parameters with the results from the complete data [20,21]. The appeal of this simulation approach is that it provides a direct comparison of the imputation procedures in terms of how well they reproduce the results that we would have observed if there had been no missing data. The approach separates the evaluation of the MI procedures from the component of variability associated with repeated sampling of the original dataset, based on the assumption that the complete-data estimate (and associated variance estimate) is valid for the complete-data sampling model. A limitation of the approach is that conclusions strictly only apply to the particular dataset used for the simulation experiments. However, our results reveal clear differences between the imputation methods that seem likely to be generalizable to other datasets.

A further limitation of this study is the restriction to imputations conducted in a univariate setting. However, since this is the first study to present a comprehensive comparison of a range of approaches to handling limited-range variables it was important to begin with a simple example to ensure that possible influences on the performance of the imputation models were kept to a minimum. We do, however, recommend further testing of these imputation approaches in datasets that have multiple incomplete variables, which will require the use of MVN imputation or MICE rather than univariate imputation models.

## Conclusions

The findings of the current study suggest the best method to impute limited-range variables is to impute on the raw scale with no restrictions to the range, and with no post-imputation rounding, as previously recommended by von Hippel [14]. Although this imputation method results in some implausible values, it appears to be the most consistent method with low bias and reliable coverage in repeated sampling of missingness, irrespective of the amount of skewness in the data.

## Abbreviations

GHQ: General Health Questionnaire; MAR: Missing at random; MCAR: Missing completely at random; MI: Multiple imputation; MICE: Multivariate imputation with chained equations; MVN: Multivariate normal; VAHCS: Victorian Adolescent Health Cohort Study.

## Competing interests

The authors declare they have no competing interests.

## Authors’ contributions

LR conceived of the study, performed the simulations and analyses and wrote the first draft of the paper. KJL, HR and JBC participated in the design of the study and provided input into the interpretation of the results. All authors participated in the drafting of the manuscript and read and approved the final manuscript.

## Pre-publication history

The pre-publication history for this paper can be accessed here:

http://www.biomedcentral.com/1471-2288/14/57/prepub

## Supplementary Material

Additional file 1 Performance measures for the estimation of the regression coefficient with GHQ scores on transformed scale. Click here for file
