# Supplementary material for: Comparison of methods for imputing limited-range variables: a simulation study
Source: BMC Med Res Methodol. 2014 Apr 26;14:57. doi: 10.1186/1471-2288-14-57 (PMC4021274; doi:10.1186/1471-2288-14-57)
Supplement: Additional file 1 — Performance measures for the estimation of the regression coefficient with GHQ scores on transformed scale. [file 1471-2288-14-57-S1.docx]

**Additional file 1: Table S1.** Performance measures for the estimation of the regression coefficient with GHQ scores on transformed scale

| **Likert** | $\hat{\boldsymbol{Q}}\boldsymbol{=}0.03227$ | | $\boldsymbol{U=}0.02143$ |  |  |  |
| --- | --- | --- | --- | --- | --- | --- |
| **MCAR** | $\boldsymbol{E[}{\bar{\boldsymbol{Q}}}_{\boldsymbol{m}}\boldsymbol{]}$ | **bias** | $\boldsymbol{E[}{\bar{\boldsymbol{U}}}_{\boldsymbol{m}}\boldsymbol{]}$ | $\boldsymbol{Var(}{\bar{\boldsymbol{Q}}}_{\boldsymbol{m}}\boldsymbol{)}$ | $\left( \boldsymbol{1+}\boldsymbol{m}^{\boldsymbol{-1}} \right)\boldsymbol{E[}\boldsymbol{B}_{\boldsymbol{m}}\boldsymbol{]}$ | **coverage for** $\hat{\boldsymbol{Q}}$ |
| regression, non-rounded | 0.02756 | -0.00471 | 0.02183 | 0.00025 | 0.00022 | 0.936 |
| post-imputation rounding | 0.02768 | -0.00459 | 0.02189 | 0.00025 | 0.00021 | 0.932 |
| truncated regression | 0.02831 | -0.00397 | 0.02208 | 0.00025 | 0.00021 | 0.923 |
| predictive mean matching | 0.02416 | -0.00811 | 0.02224 | 0.00018 | 0.00016 | 0.910 |
| **MAR** |  |  |  |  |  |  |
| regression, non-rounded | 0.02089 | -0.01138 | 0.02265 | 0.00076 | 0.00065 | 0.936 |
| post-imputation rounding | 0.01715 | -0.01512 | 0.02292 | 0.00087 | 0.00068 | 0.921 |
| truncated regression | 0.02205 | -0.01022 | 0.02287 | 0.00078 | 0.00063 | 0.929 |
| predictive mean matching | 0.01489 | -0.01738 | 0.02310 | 0.00037 | 0.00036 | 0.899 |
|  |  |  |  |  |  |  |
| **C-GHQ** | $\hat{\boldsymbol{Q}}\boldsymbol{=}0.04794$ | | $\boldsymbol{U=}0.03967$ |  |  |  |
| **MCAR** | $\boldsymbol{E[}{\bar{\boldsymbol{Q}}}_{\boldsymbol{m}}\boldsymbol{]}$ | **bias** | $\boldsymbol{E[}{\bar{\boldsymbol{U}}}_{\boldsymbol{m}}\boldsymbol{]}$ | $\boldsymbol{Var(}{\bar{\boldsymbol{Q}}}_{\boldsymbol{m}}\boldsymbol{)}$ | $\left( \boldsymbol{1+}\boldsymbol{m}^{\boldsymbol{-1}} \right)\boldsymbol{E[}\boldsymbol{B}_{\boldsymbol{m}}\boldsymbol{]}$ | **coverage for** $\hat{\boldsymbol{Q}}$ |
| regression, non-rounded | 0.04333 | -0.00461 | 0.03832 | 0.00066 | 0.00076 | 0.960 |
| post-imputation rounding | 0.04544 | -0.00249 | 0.03974 | 0.00072 | 0.00073 | 0.952 |
| truncated regression | 0.03990 | -0.00804 | 0.04112 | 0.00081 | 0.00073 | 0.932 |
| predictive mean matching | 0.03680 | -0.01114 | 0.04030 | 0.00050 | 0.00057 | 0.941 |
| **MAR** |  |  |  |  |  |  |
| regression, non-rounded | 0.03821 | -0.00973 | 0.03851 | 0.00184 | 0.00206 | 0.964 |
| post-imputation rounding | 0.04001 | -0.00793 | 0.04008 | 0.00198 | 0.00205 | 0.956 |
| truncated regression | 0.05837 | 0.01043 | 0.04113 | 0.00237 | 0.00202 | 0.927 |
| predictive mean matching | 0.02423 | -0.02371 | 0.04084 | 0.00101 | 0.00119 | 0.921 |
|  |  |  |  |  |  |  |
| **Standard** | $\hat{\boldsymbol{Q}}\boldsymbol{=}0.05236$ | | $\boldsymbol{U=}0.04202$ |  |  |  |
| **MCAR** | $\boldsymbol{E[}{\bar{\boldsymbol{Q}}}_{\boldsymbol{m}}\boldsymbol{]}$ | **bias** | $\boldsymbol{E[}{\bar{\boldsymbol{U}}}_{\boldsymbol{m}}\boldsymbol{]}$ | $\boldsymbol{Var(}{\bar{\boldsymbol{Q}}}_{\boldsymbol{m}}\boldsymbol{)}$ | $\left( \boldsymbol{1+}\boldsymbol{m}^{\boldsymbol{-1}} \right)\boldsymbol{E[}\boldsymbol{B}_{\boldsymbol{m}}\boldsymbol{]}$ | **coverage for** $\hat{\boldsymbol{Q}}$ |
| regression, non-rounded | 0.00268 | -0.04968 | 0.01672 | 0.00006 | 0.00025 | 0.114 |
| post-imputation rounding | 0.03925 | -0.01311 | 0.03827 | 0.00057 | 0.00084 | 0.977 |
| truncated regression | 0.04318 | -0.00918 | 0.04317 | 0.00100 | 0.00081 | 0.923 |
| predictive mean matching | 0.03890 | -0.01346 | 0.04338 | 0.00062 | 0.00064 | 0.940 |
| **MAR** |  |  |  |  |  |  |
| regression, non-rounded | 0.00372 | -0.04864 | 0.01567 | 0.00012 | 0.00051 | 0.346 |
| post-imputation rounding | 0.03314 | -0.01922 | 0.03888 | 0.00146 | 0.00222 | 0.989 |
| truncated regression | 0.08952 | 0.03716 | 0.04173 | 0.00212 | 0.00205 | 0.857 |
| predictive mean matching | 0.02661 | -0.02575 | 0.04478 | 0.00104 | 0.00137 | 0.964 |

*Key*: $\hat{Q}=$complete data estimate; $U\boldsymbol{=}$ estimated variance of $\hat{Q}$ from complete data; $E[\bar{Q}_{m}]$ = average of MI-based point estimates across 1000 simulated datasets; bias = difference between $E\left[ \bar{Q}_{m} \right]$ and $\hat{Q}$; $E[\bar{U}_{m}]$ = average of estimated within-imputation variance across simulated datasets; $Var(\bar{Q}_{m})$ = variance of the MI point estimates across simulated datasets; $\left( 1+m^{-1} \right)E[B_{m}]$ = average of estimated between-imputation variance (with adjustment for number of imputations) across simulated datasets; coverage = proportion of (nominally) 95% confidence intervals that contain the complete data estimate.
